# Dermoscopic Features of Different Forms of Cutaneous Mastocytosis: A Systematic Review

**DOI:** 10.3390/jcm11164649

**Published:** 2022-08-09

**Authors:** Martyna Sławińska, Agnieszka Kaszuba, Magdalena Lange, Roman J. Nowicki, Michał Sobjanek, Enzo Errichetti

**Affiliations:** 1 Department of Dermatology, Venereology and Allergology, Faculty of Medicine, Medical University of Gdańsk, 97-331 Gdańsk, Poland; 2 Department of Experimental and Clinical Medicine, Universita degli Studi di Udine, 33100 Udine, Italy

**Keywords:** dermoscopy, dermatoscopy, trichoscopy, mastocytosis, review

## Abstract

The term mastocytosis refers to a heterogeneous group of disorders characterised by accumulation of clonal mast cells in different organs, most commonly in the skin. Little is known about the role of dermoscopy in the diagnostics of mastocytosis. To date, no systematic review on the dermoscopic features of cutaneous mastocytosis has been performed. The aim of this study was to summarise the current knowledge in the field as well as to identify the knowledge gaps to show possible directions for further studies, based on a systematic search of PubMed, Scopus, and Web of Science databases and related references published before 3 January 2022. Dermoscopic features, type of dermoscope, polarisation mode, magnification, and number of cases were analysed. In total, 16 articles were included in this review (3 case series and 13 case reports), analysing 148 patients with different variants of cutaneous mastocytosis; all of the studies analysed had a low level of evidence (V). The main dermoscopic features of urticaria pigmentosa included brown structureless areas, brown lines arranged in a network, and linear vessels distributed in a reticular pattern, with this last finding also being typical of telangiectasia macularis eruptiva perstans. The presence of either circumscribed yellow structureless areas or diffuse yellowish background was a constant pattern of mastocytoma, while nodular, pseudoangiomatous xanthelasmoid, and plaque-type mastocytosis were typified by light-brown structureless areas and/or pigment network, though the first two variants also showed yellow/yellow-orange structureless areas. Finally, pigmented streaks of radial distribution surrounding hair follicles were described to be a pathognomonic dermoscopic feature of pseudoxanthomatous mastocytosis. Although this review shows that the various clinical forms of cutaneous mastocytosis may feature diagnostic dermoscopic clues, it also underlines the need for further investigation as several relevant data are missing, including evaluation of dermoscopic pattern according to anatomical locations or “lesion age”, studies on rare mastocytosis variants, evaluation of the prognostic role of dermoscopy in the context of systemic involvement, and comparative analyses with common clinical mimickers.

## 1. Introduction

The term mastocytosis refers to a heterogeneous group of disorders characterised by accumulation of clonal mast cells in different organs, most commonly in the skin, bone marrow, liver, spleen, and lymph nodes. Cutaneous involvement may be either the only manifestation of the disease (Cutaneous Mastocytosis, CM) or it may be associated with systemic disease (Systemic Mastocytosis, SM) [1,2,3]. In contrast to adults, CM predominates in children [4].

According to the current World Health Organization (WHO) classification, CM is divided into three forms: maculopapular cutaneous mastocytosis (MPCM) (including clinical subtypes previously known as urticaria pigmentosa [UP] and telangiectasia macularis eruptiva perstans [TMEP]), diffuse cutaneous mastocytosis (DCM), and mastocytoma of the skin [5]. Other clinical variants of CM have been described, e.g., nodular mastocytosis, plaque-type mastocytosis, pseudoangiomatous xanthelasmoid mastocytosis, and pseudoxanthomatous localised mastocytosis, though they are no longer recognised as separate entities but as clinical subtypes of either MPCM or mastocytoma based on the number of the lesions (>5 MPCM and ≤5 mastocytoma) [2,6,7,8,9,10,11,12,13]. The diagnosis of cutaneous mastocytosis is generally based on clinical assessment (presentation of cutaneous lesions, positive Darier’s sign, and symptoms arising from mediator release) in association with additional investigations, such as histopathological, immunohistochemical, and sometimes genetic assessment.

Main differential diagnoses of CM include urticaria, juvenile xanthogranuloma, arthropod bites, bullous impetigo, autoimmune bullous skin disorders, epidermolysis bullosa, staphylococcal scalded skin syndrome, and café-au-lait macules (Supplementary Table S1) [14,15]. Dermoscopy is a supportive tool in the diagnosis of different cutaneous disorders [16]. In recent years, new papers describing the dermoscopic features of mastocytosis have been published. The aim of this study was to summarise current data on the diagnostic utility of (video) dermoscopy of skin involvement in patients with mastocytosis.

## 2. Materials and Methods

A comprehensive search of the literature using the PubMed, Scopus, and Web of Science electronic databases using the keywords ‘dermoscopy’ OR ‘dermatoscopy’ OR ‘trichoscopy’ OR ‘videodermoscopy’ OR ‘videodermatoscopy’ in combination with ‘mastocytosis’ OR ‘urticaria pigmentosa’ OR ‘mastocytoma’ OR ‘teleangiectasia macularis eruptiva perstans’ was performed by two investigators (M. Sławińska., A. Kaszuba) over a time period from inception to 2 January 2022. After the initial search was performed, two reviewers independently screened titles and abstracts for inclusion and exclusion criteria. In doubtful cases, decision on inclusion was based on the opinion of the third investigator (M. So). Based on title and abstract analysis, researchers selected the articles concerning dermosopic features of mastocytosis. At this step, we excluded records not related to the topic, non-English language manuscripts, review articles, and duplicates. In the relevant articles assessed full-text, references were searched for additional records. Finally, articles not containing quantitative data on dermoscopic observations were excluded. In addition to dermoscopic features, type of dermoscope, polarisation mode, magnification, and number of cases were analysed and summarised. The Oxford 2011 Levels of Evidence was used to classify the level of evidence of each article [17]. As the analysed papers concerned diagnostic studies single case reports were labelled as level of evidence V. Additionally, corresponding terminology based on the International Dermoscopy Society consensus paper has been added [18].

## 3. Results

**Figure 1:**
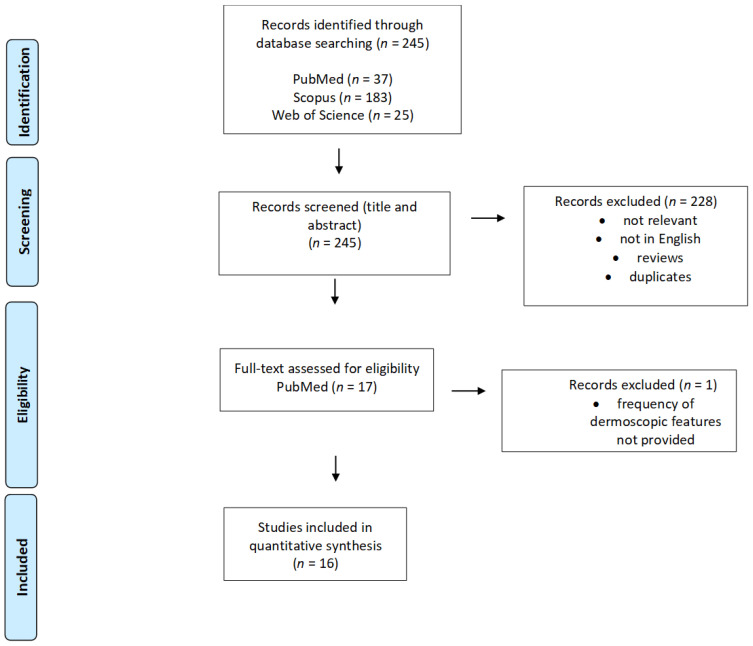
PRISMA flow diagram demonstrating the selection process for study inclusion in the systematic review.

Of the 245 records found initially in PubMed, Scopus, and Web of Science databases, a total of 17 articles were assessed full-text after title and abstract screening. Of these 17 articles, one was excluded as it did not meet the inclusion criteria, and none were included after reference screening. Thus, in total, 16 articles were included in this review (3 case series and 13 case reports), analysing 148 patients with different CM variants. The flow chart reporting the study selection process is presented in Figure 1.

The type of dermoscope used in the study was mentioned in seven records, magnification in eight (seven used ×10 magnification and one ×50 and ×200 magnification), polarisation was mentioned in five records (polarised light was applied in all of them), and information concerning the use or not of an immersion interface was not provided in any of the analysed studies.

Table 1 presents the details of the analysed studies.

### 3.1. Maculopapular Cutaneous Mastocytosis (UP Clinical Subtype)

**Figure 2:**
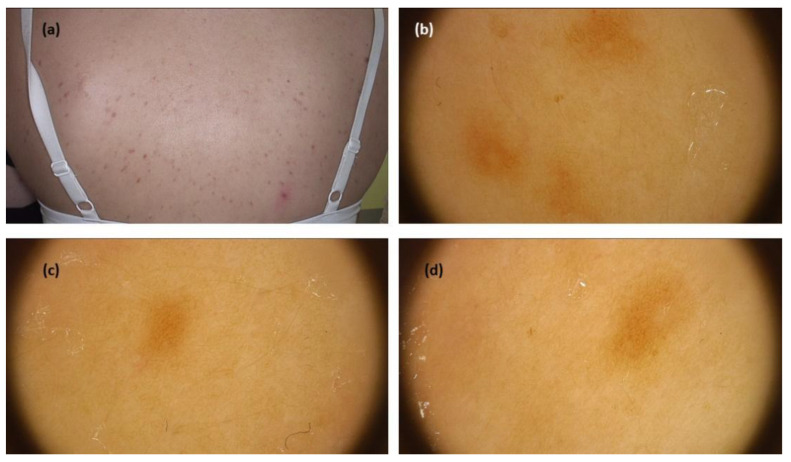
(**a**) Maculopapular cutaneous mastocytosis (urticaria pigmentosa clinical subtype)—clinical presentation. (**b**–**d**) Dermoscopy shows brown reticular lines (pigment network)(FotoFinder, Medicam 800 HD, FotoFinder Systems GmbH, Bad Birnbach, Germany; ×20 magnification, immersion gel).

The systematic review revealed 99 cases in seven studies. Most data on dermoscopy of this mastocytosis variant came from a study by Vano-Galvan et al. [9], who analysed 90 patients with MPCM. The most prevalent dermoscopic findings were light-brown blots (structureless areas) (43/90; 47.8%), pigment networks (36/90; 40.0%), and vascular patterns including linear vessels in reticular distribution—described as reticular vessels—and dotted vessels (11/90; 12.2%). Interestingly, the study showed that the vascular dermoscopic pattern was an independent predictive factor for the need for daily anti-mediator therapy. The remaining data from smaller case reports/case series (six studies, nine patients) confirmed the presence of pigment network (brown reticular lines) in all cases (in two of them associated with a red or light-brown background) [9,10,19,20,21,22,23] (Figure 2).

### 3.2. Maculopapular Cutaneous Mastocytosis (TMEP Clinical Subtype)

**Figure 3:**
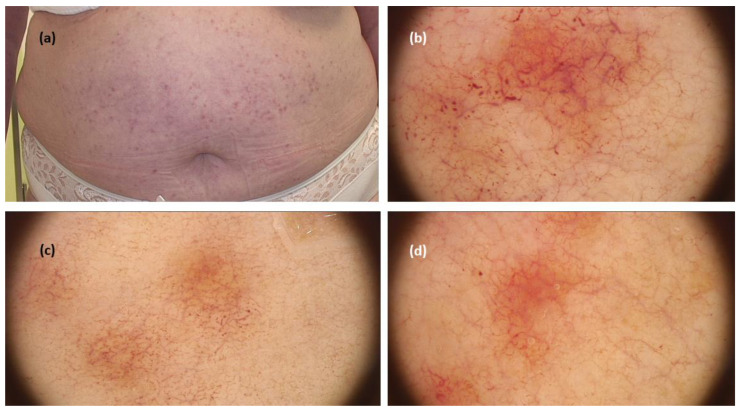
(**a**) Maculopapular cutaneous mastocytosis (telangiectasia macularis eruptiva perstans clinical subtype)—clinical presentation. (**b**–**d**). Dermoscopy shows reticular vascular pattern (thin reticular telangiectasias) over erythematous background (FotoFinder, Medicam 800 HD, FotoFinder Systems GmbH, Bad Birnbach, Germany; ×20 magnification, immersion gel).

When it comes to TMEP, a systematic review revealed 13 cases from five studies, including one case limited only to acral areas. The three main dermoscopic patterns were reticular vascular pattern (thin reticular telangiectasias) observed in all cases, in three cases associated with an erythematous background, in two cases with pigment network (brown reticular lines), and in one with brownish background [9,10,11,12] (Figure 3).

### 3.3. Mastocytoma

**Figure 4:**
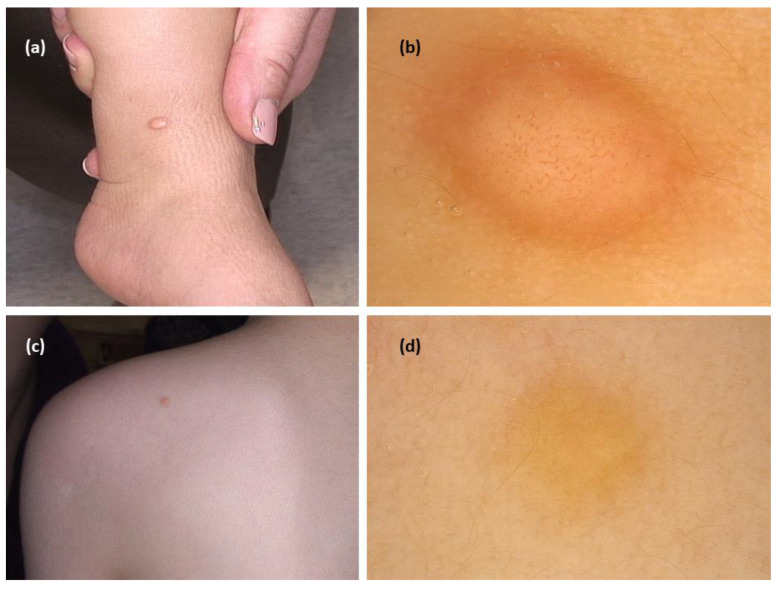
(**a**) Mastocytoma—clinical presentation. (**b**) Dermoscopy shows central polymorphic vessels and peripheral yellow brownish structureless area (FotoFinder, Medicam 800 HD, FotoFinder Systems GmbH, Bad Birnbach, Germany; ×20 magnification, immersion gel). (**c**) Mastocytoma—clinical presentation. (**d**) Dermoscopy shows yellow structureless pattern (FotoFinder, Medicam 800 HD, FotoFinder Systems GmbH, Bad Birnbach, Germany; ×20 magnification, immersion gel).

The systematic review revealed 15 cases from five studies. In all cases, the authors described the presence of yellow structureless areas (blot)/yellowish background. In most of them (11/13; 84.6%), it was the only observed pattern [9,24,25,26,27]. Additional structures were peripheral brown reticular lines (3/13), a central white structureless area (2/13), and vessels in a central distribution (vessel morphology was not described; 1/13) (Figure 4).

### 3.4. Other Clinical Forms of CM

**Figure 5:**
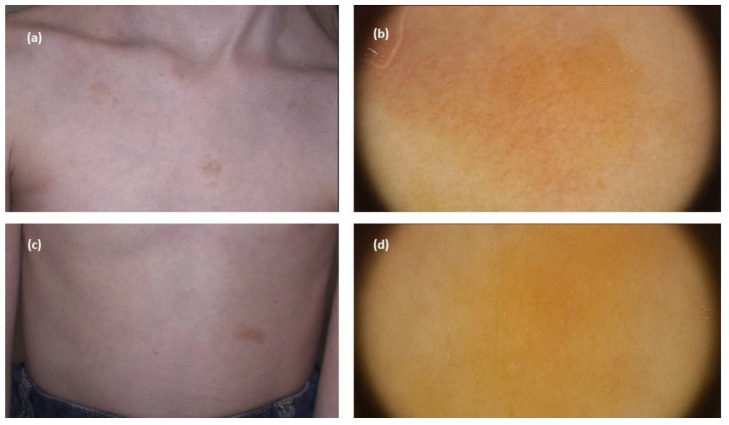
(**a**,**c**) Maculopapular cutaneous mastocytosis (plaque-type mastocytosis clinical subtype)—clinical presentation. (**b**) Dermoscopy shows reticular vascular pattern (thin reticular telangiectasias) over yellow background. (**d**) Dermoscopy shows yellow structureless areas (FotoFinder, Medicam 800 HD, FotoFinder Systems GmbH, Bad Birnbach, Germany; ×20 magnification, immersion gel).

With regard to nodular mastocytosis and plaque-type mastocytosis, Vano-Galvan et al. [9] in their study analysed eleven and eight instances of the former and latter variant, respectively. In detail, the main dermoscopic features of nodular mastocytosis were yellow-orange structureless areas (6/11; 54.6%), pigment networks (5/11; 45.5%), and light-brown structureless areas (3/11; 27.3%), while plaque-type mastocytosis was associated with light-brown structureless areas (5/8; 62.5%) and pigment networks (3/18; 37.5%) [9] (Figure 5).

Considering pseudoangiomatous xanthelasmoid mastocytosis, only one dermoscopic case of this rare entity was published revealing a pigment network (brown reticular lines) and yellow blots (structureless areas) [7].

Finally, an instance of pseudoxanthomatous localised mastocytosis involving the vulva showed pigmented streaks with radial distribution surrounding hair follicles, and this pattern was considered pathognomonic by the authors [6].

## 4. Discussion

The aim of this review was to summarise the current knowledge on the role of dermoscopy in diagnosis of mastocytosis. The analysis of published studies revealed that almost all data (127 out of 148 patients) came from one large case series, and the remaining were small case series/case reports. All of the analysed studies showed a low level of evidence (V).

Based on this review, the main dermoscopic features of UP included brown structureless areas, brown lines arranged in a network, and linear vessels distributed in a reticular pattern. The first two findings were due to basal cell layer hyperpigmentation, a typical histological feature of this form of mastocytosis, while the vascular pattern was the result of dermal vessel dilation. This last histological finding was also responsible for the main dermoscopic feature of TMEP—the so-called “vascular reticular pattern” (thin reticular telangiectasias). By contrast, the presence of either circumscribed yellow structureless areas or diffuse yellowish background was a repetitive dermoscopic pattern of mastocytoma histologically resulting from a compact mast cell infiltration of the dermis. Moving to nodular, pseudoangiomatous xanthelasmoid, and plaque-type mastocytosis, all of them may be typified by light-brown structureless areas and/or pigment network as a result of basal cell layer hyperpigmentation, yet only the first two variants may also show yellow/yellow-orange structureless areas, likely due to a denser cellular infiltration. Finally, pigmented streaks of radial distribution surrounding hair follicles were described as being a pathognomonic dermoscopic feature of pseudoxanthomatous mastocytosis, though this observation needs to be confirmed in further studies as it came from a single report [28].

Importantly, most of the remaining mentioned findings were not specific to mastocytosis and thus, should be interpreted carefully along with clinical and histopathological findings. Brown lines arranged in a network should be differentiated from melanocytic lesions or dermatofibroma, while in cases of yellow-orange structureless areas, diagnosis of juvenile xanthogranuloma, xanthoma, solitary reticulohistiocytoma, sebaceous tumours, keratin accumulation or scaly disorders, elastic fibres disorders, and others should be considered [29].

## 5. Conclusions

Despite several articles being published on the dermoscopy of CM, there is still a need for further investigations in this regard as the current evidence was from low-quality studies. Additionally, there were various aspects that have not been investigated so far, including evaluation according to anatomical locations or “lesion age”, studies on rare mastocytosis variants, evaluation of the prognostic role of dermoscopy in the context of systemic involvement, and comparative analyses with common clinical mimickers. Based on current knowledge, it seems that dermoscopy will remain a complementary technique in mastocytosis diagnosis, as potential overlap with structures observed in other entities exists.

## Figures and Tables

**Table 1:** The summary of the dermoscopic features for different forms of cutaneous mastocytosis.

Clinical Manifestation of CM	First Author, Journal, Year	Dermoscopic Features per Diagnosis (Number/%)	Corresponding Terminology Based on International Dermoscopy Society Consensus Document	Dermoscopic-Histopathological Correlation Discussed in the Article	Type of Dermoscope/Magnification	Polarisation/Immersion	Study Design	Number of Cases of Specific Mastocytosis Subtype	Level of Evidence	Aspects Important for Clinical Practice
***Maculopapular CM (MPCM)*** *urticaria pigmentosa clinical subtype*	Akay, *Dermatology*, 2008 [10]	brown reticular lines	brown lines arranged in a network-like structure	basal hyperpigmentation and increase in mast cells in the dermis	DermLite II Pro HR 3Gen/NR	NR/NR	case series	3	V	brown reticular lines seen on dermoscopy may be present also in melanocytic lesion, dermatofibroma, solar lentigo, ink-spot lentigo, seborrheic keratosis, accessory nipple
	Vano-Galvan, *Arch Dermatol*, 2011 [9]	light-brown blot (43/90;47.8%); pigment network (36/90; 40.0%); vascular pattern (11/90; 12.2%)	brown structureless areas; brown lines arranged in a network-like structure; linear vessels arranged in a reticular pattern; dotted vessels	hyperpigmentation of basal layer (mild and homogenous); mast cells in the dermis; hyperpigmentation of the basal layer (marked on the rete ridges); mast cells in the dermis; blood vessel dilatation	DermLite 3Gen/×10	NR/NR	case series	90	V	vascular pattern was an independent predictive factor for the need for daily anti-mediator therapy
	Gutiérrez-González, *Dermatol Online J*, 2011 [19]	brown reticular lines	brown lines arranged in a network-like structure	increase in melanocytes and melanin deposits in the basal layer, mast cells in the dermis	NR/NR	NR/NR	case report	1	V	papules dermoscopically mimicked melanocytic nevi
	Miller, *An Bras Dermatol*, 2013 [20]	pigment network, light-brownish blot	brown lines arranged in a network-like structure, brown structureless areas	basal cell layer hyperpigmentation, mast cells and lymphocytes in dermis	NR/×10	NR/NR	case report	1	V	
	Nirmal, *Indian Dermatol Online J*, 2019 [21]	brown reticular lines	brown lines arranged in a network-like structure	basal cell layer hyperpigmentation; mast cells in dermis	DermLite DL3,3Gen/×10	polarised/NR	case series	2	V	brownish lines seen on dermoscopy were darker in case of mastocytosis with positive Darier sign
	Chauhan, *Indian Dermatol Online J*, 2020 [22]	brown reticular lines, reddish background	brown lines arranged in a network-like structure -	basal cell layer hyperpigmentation, mild epidermal spongiosis, increase in mast cells and lymphocytes in dermis	DermLite II hybrid m; 3Gen/×10	polarised/NR	case report	1	V	reddish background was suggested by the authors as a feature helpful in differentiation between mastocytosis and melanocytic nevi
	Amorim, *Int J Dermatol*, 2021 [23]	reticular lines (pigmented network)	brown lines arranged in a network-like structure	mastocytes on superficial dermis	NR/NR	NR/NR	case report	1	V	
***MPCM*** *telangiectasia macularis eruptiva perstans clinical subtype*	Akay, *Dermatology*, 2008 [10]	thin reticular telangiectasias (3/3); erythematous background (2/3); brown reticular lines (1/3)	linear vessels arranged in a reticular pattern - brown lines arranged in a network-like structure	dilated dermal vessels; mast cells in superficial dermis; NR	DermLite II Pro HR 3Gen/NR	NR/NR	case series	3	V	reticular vascular pattern may be helpful in differentiation between TMEP and other eruptions
	Vano-Galvan, *Arch Dermatol*, 2011 [9]	reticular vascular pattern (7/7; 100%)	linear vessels arranged in a reticular pattern	dilation of the blood vessels	DermLite 3Gen/×10	NR/NR	case series	7	V	
	Unterstell, *An Bras Dermatol*, 2013 [12]	thin and tortuous linear vessels, mild erythema, fine pigment network	linear-curved vessels - brown lines arranged in a network-like structure	dilatation and vascular proliferation associated with the presence of mast cells in the dermis, NR	NR/NR	NR/NR	case report	1	V	
	Kumar, *Indian Dermatol Online J*, 2019 [11]	reticular vascular pattern of linear and branching vessels, brownish background	linear vessels and linear vessels with branches in a reticular distribution, brown structureless areas	dilated superficial capillaries surrounded by mast cells in the papillary dermis; NR	Dino Lite AM413ZT Digital Microscope/×50	polarised/NR	case report	1	V	at higher magnification (200×), branching vessels encircled the eccrine glands (visible as white dots)
***MPCM*** *telangiectasia macularis eruptiva perstans clinical subtype—limited to acral areas*	Sammut, *Int J Dermatol*, 2019 [8]	reticular vascular pattern	linear vessels arranged in a reticular pattern	prominent ectatic blood vessels in the upper and mid dermis	NR/NR	NR/NR	case report	1	V	
***Mastocytoma***	Vano-Galvan, *Arch Dermatol*, 2011 [9]	yellow-orange blot (11/11; 100%)	yellow-orange structureless areas	dense infiltration of mast cells along the papillary and reticular dermis	DermLite 3Gen/×10	NR/NR	case series	11	V	in differential diagnosis of lesions presenting with yellow-orange structureless pattern on dermoscopy consider: juvenile/adult xanthogranuloma, sebaceous hyperplasia, reticulohistiocytoma, xanthomatous dermatofibroma
	Adya, *Indian Dermatol Online J*, 2018 [24]	central white structureless area, yellow background, peripheral brown reticular lines	central white structureless area, diffuse yellow structureless area, brown lines arranged in a network-like structure	accumulation of serosanguineous fluid produced due to excoriation of the epidermis in the centre of the lesion, diffuse mononuclear cell infiltrate involving the dermis extending into and expanding the dermal papillae, increased melanisation of the basal layer	DermLite DL3/×10	polarised/NR	case report	1	V	dermoscopy is helpful in differentiation with juvenile xanthogranuloma
	Gündüz, *Dermatol Pract Concept*, 2019 [25]	central vascular structures * peripheral yellow structureless area	- peripheral yellow structureless area	central vascular structure might reflect the detachment of epidermis due to the bullous reaction, dense mast cell infiltration in dermis	NR/×10	NR/NR	case report	1	V	
	Kumar, *BMJ Case Reports*, 2020 [26]	central whitish area, reticulate light brown rim, yellowish background	central white structureless area, brown lines arranged in a network-like structure, diffuse yellow structureless area	NR, basal layer melanisation, diffuse infiltration of the superficial epidermis by mastocytes	NR/NR	NR/NR	case report	1	V	
	Mukherje, *Dermatol Pract Concept*, 2021 [27]	pigment network, yellow background	brown lines arranged in a network-like structure, diffuse yellow structureless area	basal layer hyperpigmentation, mast cells in dermis	DermLite DL4/×10	polarised/NR	case report	1	V	provocation of Darier sign on dermoscopy shows decrease in yellow colour and pigment network intensity with appearance of peripheral erythema
***Other clinical forms*** *Plaque-type mastocytosis*	Vano-Galvan, *Arch Dermatol*, 2011 [9]	light-brown blot (5/8; 62.5%); pigment network (3/18; 37.5%)	brown structureless areas; brown lines arranged in a network-like structure	hyperpigmentation of basal layer (mild and homogenous), mast cells in the dermis; hyperpigmentation of the basal layer (marked on the rete ridges), mast cells in the dermis	DermLite 3Gen/×10	NR/NR	case series	8	V	
***Other clinical forms*** *Nodular mastocytosis*	Vano-Galvan, *Arch Dermatol*, 2011 [9]	light-brown blot (3/11; 27.3%); pigment network (5/11; 45.5%); yellow-orange blot (6/11; 54.6%)	brown structureless areas; brown lines arranged in a network-like structure; yellow-orange structureless areas	hyperpigmentation of basal layer (mild and homogenous), mast cells in the dermis; hyperpigmentation of the basal layer (marked on the rete ridges), mast cells in the dermis; dense infiltration of mast cells along the papillary and reticular dermis	DermLite 3Gen/×10	NR/NR	case series	11	V	
***Other clinical forms*** *Pseudoangiomatous xanthelasmoid mastocytosis*	Salah, *J Eur Acad Dermatol*, 2016 [7]	brown reticular pattern, yellow blots	brown lines arranged in a network-like structure. yellow structureless areas	patchy basal hyperpigmentation, mast cells in dermis	NR/NR	NR/NR	case report	1	V	main clinical differential diagnoses: xanthoma, vascular tumour/malformation, xanthogranuloma
***Other clinical forms*** *Pseudoxanthomatous localised mastocytosis*	Li, *An Bras Dermatol*, 2018 [6]	pigmented stripes radiating from hair follicles, pink background, linear branched vessels, reticular vessels	specific clue, - linear vessels with branches, linear vessels arranged in a reticular pattern	hyperpigmentation of keratinocytes in the basal layer, dense infiltration of mast cells, NR, NR	NR/NR	NR/NR	case report	1	V	vulva may be predilection site for localised cases; clinical differential diagnoses include pseudoxanthoma elasticum, juvenile xanthogranuloma, xanthoma; pigmented lines arranged radially around hair follicles may be a specific feature

NR—not reported; *—vessel morphology not provided.

## Data Availability

Not applicable.

## Supplementary Materials

The following supporting information can be downloaded at: https://www.mdpi.com/article/10.3390/jcm11164649/s1, Table S1: Dermoscopic features of the entities that may clinically resemble cutaneous mastocytosis. References [30,31,32,33,34,35,36,37,38,39,40,41,42,43,44] are cited in the supplementary materials.
