# Supplementary material for: Dermoscopic Features of Different Forms of Cutaneous Mastocytosis: A Systematic Review
Source: J Clin Med. 2022 Aug 9;11(16):4649. doi: 10.3390/jcm11164649 (PMC9410418; doi:10.3390/jcm11164649)
Supplement: Supplementary file 1 [file jcm-11-04649-s001.zip › jcm-1801135-supplementary.pdf]

Supplementary Table S1

Dermoscopic features of the entities that may clinically resemble cutaneous mastocytosis.

Diagnosis	Dermoscopic features
Urticaria	Common urticaria:<br>● prominent sometimes reticular red lines<br>● structureless avascular areas (vessels obscured by prominent oedema)<br><br>Urticarial vasculitis:<br>● purpuric/red dots or globules<br>● orange-brown background [1][2]
Juvenile xanthogranuloma	● "setting sun" pattern,<br>● yellow/orange–pink/red colour<br>● yellow globules<br>● shiny white streaks<br>● irregularly distributed types of vascular structures (most common vessel types: dotted, linear, and branching–arboriform)<br>● less common: brown globules, pale-brown network, negative network, erosion/ulceration, rosettes, and haemorrhage [3][4][5]
Arthropod bites/infestations	Scabies:<br>● whitish scales<br>● reddish background<br>● dark-brown triangular structures located at the end of whitish structureless / wavy lines (delta-wing jets with contrail)<br>● brown triangular structure alone ('delta glider', 'hang-glider', 'circumflex accent-like' and 'delta-wing' sign (classic scabies and nodular scabies)), 'grey-edged line' sign (classic scabies), wake sign (classic scabies), erythema, and multiple burrows in noodle-like fashion (crusted scabies) [6][7][8][9]<br><br>Bed bugs:<br>● diffuse erythema<br>● haemorrhagic clod(s)(bite spot)<br>● telangiectasias<br>● pinkish background [8][9]<br><br>Wasp sting:<br>● detection of stingers (linear brown foreign bodies) [9]
Bullous impetigo	● brown–yellow crusts<br>● superficial erosions<br>● collarettes of scale [10]
Autoimmune bullous skin disorders	Pemphigus vulgaris:

	• lesion surface: yellowish–pink translucent area, no pigment network, central brown area; erythematous background<br>• erosive areas with irregular and/or angulated borders<br>• peripheral epidermal remnants [11][12][13]<br><br>Bullous pemphigoid:<br><br>• lesion surface: yellowish–pink translucent areas, distorted pigment network<br>• prominent follicular and eccrine openings<br>• perifollicular and perieccrine pigmentation<br>• erythematous background [12]
Epidermolysis bullosa	• white rosettes (keratin blockage)<br>• white lacy network<br>• white globules<br>• dark brown dots and globules<br>• patchy pigment network<br>• erythematous background [12]
Staphylococcal scalded skin syndrome	• erosive areas with peripheral epidermal remnants with little leachate [13][14]
Café-au-lait macules	• light brown reticular pattern<br>• follicular sparing<br>• dark brown dots<br>• perifollicular accentuation [15]

References for Supplementary Table S1

1. Vázquez-López, F.; Fueyo, A.; Sánchez-Martín, J.; Pérez-Oliva, N. Dermoscopy for the Screening of Common Urticaria and Urticaria Vasculitis. *Arch. Dermatol.* **2008**, *144*, 568–568.
2. Suh, K.; Kang, D.; Lee, K.; Han, S.; Park, J.; Kim, S.; Jang, M. Evolution of urticarial vasculitis: A clinical, dermoscopic and histopathological study. *J. Eur. Acad. Dermatol. Venereol.* **2013**, *28*, 674–675.
3. Peruilh-Bagolini, L.; Silva-Astorga, M.; Martín, M.J.H.S.; Manoli, M.-S.; Papageorgiou, C.; Apalla, Z.; Lallas, A. Dermoscopy of Juvenile Xanthogranuloma. *Dermatology* **2020**, *237*, 946–951.
4. Di Brizzi, E.V.; Moscarella, E.; Scharf, C.; Argenziano, G.; Piccolo, V. Dermoscopy of juvenile xanthogranuloma: A retrospective descriptive study on 35 paediatric patients. *J. Eur. Acad Dermatol. Venereol.* **2022**. https://doi.org/10.1111/jdv.18316.
5. Palmer, A.; Bowling, J. Dermoscopic appearance of juvenile xanthogranuloma. *Dermatology* **2007**, *215*, 256–259.
6. Zalaudek, I.; Argenziano, G.; Di Stefani, A.; Ferrara, G.; Marghoob, A.A.; Hofmann-Wellenhof, R.; Soyer, H.P.; Braun, R.; Kerl, H. Dermoscopy in general dermatology. *Dermatology* **2006**, *212*, 7–18.
7. Errichetti, E.; Stinco, G. Dermatoscopy in general dermatology: A practical overview. *Dermatol. Ther.* **2016**, *6*, 471–507.
8. Bosseila, M.; Sonthalia, S.; Agrawal, M.; Bhatia, J.; Zeeshan; Elsamanoudy, S.; Tiwary, P.; Bhat, Y.J.; Jha, A. Entodermoscopy update: A contemporary review on dermoscopy of cutaneous infections and infestations. *Indian Dermatol. Online J.* **2021**, *12*, 220–236.
9. Chauhan, P.; Jindal, R.; Errichetti, E. Dermoscopy of skin parasitoses, bites and stings: A systematic review of the literature. *J. Eur. Acad. Dermatol. Venereol.* **2022**. https://doi.org/10.1111/jdv.18352.
10. Brazel, M.; Desai, A.; Are, A.; Motaparthi, K. Staphylococcal Scalded Skin Syndrome and Bullous Impetigo. *Medicina* **2021**, *57*, 1157.
11. Guida, S.; Longhitano, S.; Ardigò, M.; Pampena, R.; Bs, S.C.; Bigi, L.; Mandel, V.D.; Msc, C.V.; Manfredini, M.; Pezzini, C.; et al. Dermoscopy, confocal microscopy and optical coherence tomography features of main inflammatory and autoimmune skin diseases: A systematic review. *Australas. J. Dermatol.* **2021**, *63*, 15–26.
12. Narkhede, N.D.; Nikham, B.; Jamale, V.; Hussain, A.; Kale, M. Evaluation of Dermoscopic Patterns of Vesiculobullous Disorders. *Indian J. Dermatol.* **2021**, *66*, 445.
13. Errichetti, E.; Stinco, G. Dermatoscopy in life-threatening and severe acute rashes. *Clin. Dermatol.* **2019**, *38*, 113–121.
14. Miyashita, K.; Ogawa, K.; Iioka, H.; Miyagawa, F.; Okazaki, A.; Kobayashi, N.; Asada, H. Adult case of staphylococcalscalded skin syndrome differentiated from toxic epidermal necrolysis withthe aid of dermatoscopy. *J. Dermatol.* **2016**, *43*, 842–843.

15. Amatya, B. Evaluation of Dermoscopic Features in Facial Melanosis with Wood Lamp Examination. *Dermatol. Pract. Concept.* **2022**, *12*, e2022030.
